# Holothurian Glycosaminoglycan Inhibits Metastasis and Thrombosis via Targeting of Nuclear Factor-κB/Tissue Factor/Factor Xa Pathway in Melanoma B16F10 Cells

**DOI:** 10.1371/journal.pone.0056557

**Published:** 2013-02-21

**Authors:** Yang Zhao, Daohai Zhang, Sheng Wang, Li Tao, Aiyun Wang, Wenxing Chen, Zhijie Zhu, Shizhong Zheng, Xiang Gao, Yin Lu

**Affiliations:** 1 Department of Clinical Pharmacy, College of Pharmacy, Nanjing University of Chinese Medicine, Nanjing, People’s Republic of China; 2 Department of Pathology, School of Medical Sciences, University of Sydney, Sydney, New South Wales, Australia; 3 Jiangsu Key Laboratory of Efficacy and Safety Evaluation of Traditional Chinese Medicine, College of Pharmacy, Nanjing University of Chinese Medicine, Nanjing, People’s Republic of China; 4 Model Animal Research Center of Nanjing University, Nanjing, People’s Republic of China; Istituto Superiore di Sanità, Italy

## Abstract

Holothurian glycosaminoglycan (hGAG) is a high-molecular-weight form of fucosylated chondroitin sulfate and has an antithrombotic effect. Our previous studies demonstrated that hGAG efficiently inhibited tumor cell metastasis. The interplays between thrombosis and tumor progression may have a major impact on hematogenous metastasis. In this study, we demonstrated that the mouse melanoma B16F10 cells treated with hGAG displayed a significant reduction of metastasis and coagulation capacity *in vitro* and *in vivo*. Mechanistic studies revealed that hGAG treatment in B16F10 cells remarkably inhibited the formation of fibrin through attenuating the generation of activated Factor Xa (FXa), without affecting the expression of urokinase (uPA) and plasminogen activator inhibitor 1 (PAI-1) that involved in fibrinolysis. Moreover, hGAG treatment downregulated the transcription and protein expression of tissue factor (TF). Promoter deletions, site mutations and functional studies identified that the nuclear transcription factor NF-κB binding region is responsible for hGAG-induced inhibition of TF expression. While the hGAG treatment of B16F10 cells was unable to inhibit NF-κB expression and phosphorylation, hGAG significantly prevented nuclear translocation of NF-κB from the cytosol, a potential mechanism underlying the transcriptional suppression of TF. Moreover, hGAG markedly suppressed the activation of p38MAPK and ERK1/2 signaling pathways, the central regulators for the expression of metastasis-related matrix metalloproteinases (MMPs). Consequently, hGAG exerts a dual function in the inhibition of metastasis and coagulation activity in mouse melanoma B16F10 cells. Our studies suggest hGAG to be a promising therapeutic agent for metastatic cancer treatment.

## Introduction

Cancer cell metastasis is a leading cause of mortality in cancer patients. Currently, developing novel therapeutic strategies to inhibit cancer cell metastasis and eradicate residual circulating cancer cells (CTCs) in blood and the disseminated tumor cells (DTCs) in bone marrow is of paramount importance to prevent disease recurrence. CTCs develop a mechanism for survival to escape the immunosurvillence and may have a role in developing the cancer-related thrombosis. In fact, cancer cells contribute to the hypercoagulable state associated with cancer [1], [2] and this hypercoagulable state facilitates the aggressiveness of cancer cells [3]. Tissue factor (TF) is the physiological initiator of coagulation and its expression has been shown to correlate with metastatic potential [4], [5]. Moreover, the fact that addition of metastatic cancer cells to blood or plasma promotes coagulation in a TF- and phosphatidylserine (PS)-dependent manner [6]–[8] suggests a key role of TF and PS in mediating the interaction between the CTCs and the coagulation system and in promoting hematogenous metastasis.

Cancer cell metastasis is a multistep process and regulated by a complex signaling network. Accumulating evidence has demonstrated that the PI3K/p-Akt pathway modulates cell metastasis and disease progress in various tumors [9], [10]. In addition, the three major mitogen-activated protein kinases (MAPKs) family members including c-Jun N-terminal kinase (JNK), extracellular signal-regulated kinase 1 and 2 (ERK1/2) and p38 MAPK, also mediate metastasis [11], [12]. One of the important downstream cascades regulated by both PI3K and MAPKs is a pathway related to matrix metalloproteinase (MMPs) expression and secretion that is initiated by the nuclear factor-kappa B (NF-κB) [13]–[16]. Generally, NF-κB forms a complex with an inhibitor of NF-κB (IκB) and is maintained in the cytoplasm. Dissociation and nuclear translocation of NF-κB facilitate cell proliferation, angiogenesis and metastasis, leading to aggressiveness of tumors [17]–[19]. Therefore, targeting NF-κB may be beneficial for suppressing metastasis [20], [21].

**Figure 1:**
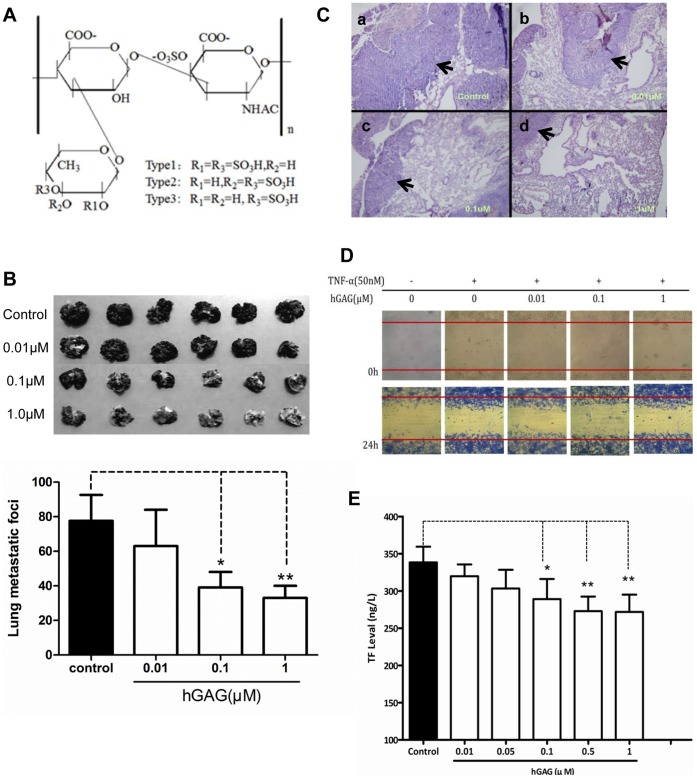
Effects of hGAG on aggressiveness of B16F10 tumor cells. (A) Chemical structure of hGAG. (B) Representative metastatic nodules on lung tissues. B16F10 tumor cells were treated with hGAG at the indicated concentration for 24 hours/37°C and injected into C57BL/6J mice through the tail vein. After 23 days, mice were sacrificed and metastatic nodules on lung surface were photographed. Metastatic nodules were counted under a dissecting microscope. Values are expressed as the mean ± SD. hGAG treatment reduces *in vivo* metastatic capacity of B16F10 tumor cells in mice. (C) Representative HE staining of lung tissue sections. Paraffin-embedded formalin-fixed lung tissues from each group were prepared and the sections were H&E stained. Arrows indicated tumor cells on each section. (D) Wound healing. B16F10 monolayer cells at 90–95% confluence were serum starved for 24 h and then carefully wounded using sterilized pipette tips (t = 0 h). After removing detached cells, cells were incubated with medium, tumor necrosis factor (TNFα, final conc. 50 nM), or TNFα in combination with hGAG at the indicated concentration for 24 h at 37°C and photographed immediately (t = 24 h). (E) TF levels in the plasma from mice assessed by ELISA. hGAG-treated B16F10 tumor cells injected into mice produced reduced level of TF. Data was expressed as mean ± S.E. (3–5 independent experiments). *p<0.05, **p<0.01.

Holothurian glycosaminoglycan (hGAG) is a high-molecular-weight fucosylated chondroitin sulfate (for structure, see Fig.1A) isolated from the sea cucumber *Stichopus japonicus* [22]. Recent studies have demonstrated its antithrombotic efficacy in mouse models with thrombin-induced pulmonary thromboembolism [23] and rat models with thrombin-induced venous thrombosis [24]. Importantly, hGAG showed a remarkable inhibitory effect on tumor metastasis *in vivo* and *in vitro* [25], indicating its potential role in anti-metastasis for cancer therapy. Because heparin is effective in the prevention and treatment of thromboembolic events in cancer patients with recurrent thrombosis due to the impact of cancer cells and chemotherapy or radiotherapy on the coagulation cascade [26]–[28], the structural similarity of hGAG with heparin may suggest a similar function and mechanism of action in terms of anti-tumor and antithrombotic capacity. Considering that hGAG is a natural product extracted from the daily consumed sea cucumber [22], hGAG is becoming a promising candidate anti-tumor agent. However, much less is known about the mechanisms that hGAG exerts on mediating the inhibition of both cancer cell metastasis and thrombosis.

This study was undertaken to investigate the effects of hGAG on the interactive system between coagulation process and tumor cell metastasis using mouse B16F10 melanoma cell line as a model. The *in vivo* studies demonstrated that the B16F10 cells treated with hGAG showed a significant reduction of metastatic capacity and a remarkable decrease of thrombosis induced by these tumor cells in a dose-dependent manner. In addition, hGAG treatment resulted in downregulation of TF and decreased the generation of activated Factor Xa (FXa), leading to a reduced cancer cell-initiated coagulation. Our studies have demonstrated that hGAG is a potential anti-tumor and anti-thrombosis agent by targeting MAPK/NF-κB/TF pathway.

## Materials and Methods

### Chemicals and Reagents

Holothurian glycosaminoglycan (hGAG) was purchased from Hualikang Biotechnology Co., Ltd (Changzhou, China). Chromogenic substrate S2222 [Bz-Ile-Glu(γ-OR)-Gly -Arg-pNA.HCl] was obtained from Chromagenix (Milano, Italy). Protein kinase C (PKC) agonist, phorbol 12-myristate 13-acetate (PMA), and p38MAPK agonist, P79350, were both from Invitrogen (Camarillo, CA). Fluo-4AM was purchased from Dojindo Company (Shanghai, China). The primary antibodies used include: TF from R&D Systems (Minneapolis, MN); p38MAPK, phosphor-p38MAPK (p-p38), Jak, p-Jak, Stat3, p-Stat3, GSK3β, p-GSK3β, p70S6K1, p-p70S6K1, and p-IκBα from Cell Signaling Technology (Danvers, MA); ERK and p-ERK from Bioworld (Minneapolis, MN); JNK, p-JNK, NF-κB p65, NF-κB p50, IκBα, Smad2, p-smad2, FAK and p-FAK were purchased from Santa Cruz Biotechnology (Santa Cruz, CA).

### Cell Culture

Mouse melanoma cells (B16F10) were obtained from American Type Culture Collection (Manassas, VA), and cultured in DMEM medium (Invitrogen, Carlsbad, CA) supplemented with 10% fetal bovine serum (Sijiqing Company Ltd., Hangzhou, China), 100 U/ml penicillin, and 100 µg/ml streptomycin in a humidified chamber at 37°C/5% CO_2_ and were used not more than 15–20 passages after the initiation of cultures.

### Metastasis Model and Coagulation Assay *in vivo*

Experimental metastasis was performed as previously described [29]. Briefly, the B16F10 tumor cells were treated for 24 h/37°C with hGAG at 0.01 µM, 0.1 µM and 1 µM, and then approximately 5×10^4^ cells were injected into the C57BL/6J mice through tail veins (6 mice per group). Mice injected with untreated cells were used as control. Twenty-three days post-injection, lung tissues were excised and melanic nodules were photographed. Metastasis was quantitatively evaluated by counting the number of pulmonary tumor nodules on the lung surface under a dissecting microscope. During the metastatic process, the blood samples were collected through orbit on day 7, day 14 and day 23, and activated partial thromboplastin time (APTT), prothrombin time (PT) and thrombin time (TT) were measured using the collected plasma as previously described [30]. The level of tissue factor (TF) in the blood samples was measured by ELISA using Mouse ELISA kit (R&D Systems, Minneapolis, MN). All the animal experiments were carried out according to the guidelines on the care and use of animals for scientific research. The protocol was reviewed and approved by the Institutional Animal Care and Use Committee of the Nanjing University of Chinese Medicine.

### *In vitro* Pro-coagulation Assay

Pro-coagulation activity was assessed using the tilt tube plasma clotting assay [31] with minor modifications. B16F10 cells at approximately 80% confluence was treated with conditioned medium containing hGAG (0–1.0 µM) for 24 h/37°C, the conditioned medium was then collected and briefly centrifuged for 5 min at 5,000 rpm to remove the cell debris. A 200 µL of conditioned medium was mixed with 200 µL of citrated normal mouse plasma, followed by addition of 200 µL of 25 mM CaCl_2_ to the tubes to initiate the clotting process. The conditioned medium from the cells treated with medium only was used as control. The clotting time was recorded to evaluate pro-coagulation activity when the semisolid gel was formed during tube tilting [31].

### ELISA

The levels of secreted uPA and PAI-1 were measured using Mouse ELISA kit (R&D Company) as per standard protocol. In brief, the B16F10 cells at approximately 80% confluence were incubated with the conditioned medium containing hGAG (0–1.0 µM) for 24 h/37°C. A 50 µl of the conditioned medium was then transferred into 96-microwells precoated with the uPA and PAI-1 antibody and incubated for 30 min at 37°C, followed by 4×washes with PBS and incubation with Biotinylated-anti-uPA or Biotinylated-anti-PAI-1 for 60 min at 37°C. The amounts of uPA and PAI-1 were determined by measuring the absorbance at 450 nm of reaction solutions containing TMB substrate. The conditioned medium from the cells treated with medium only was used as a control.

### Generation of Activated Factor Xa (FXa)

FXa generation was assessed as previously described with minor modifications [32]. Briefly, after the B16F10 tumor cells at ∼80% confluence were treated with hGAG (0–1.0 µM) for 24 h/37°C, the conditioned medium were collected and filtered to remove the cell debris. Then, 6 µL of 250 mM CaCl_2_ solution containing 5 µM prothrombin was mixed with 294 µl of the filtered conditioned medium, followed by adding 50 µl of FXa chromogenic substrate S2222 [Bz-Ile-Glu(γ-OR)-Gly-Arg-pNA.HCl] (3 mM in stock). After 2 hours incubation at 37°C, the conversion of S2222 substrate to a chromogenic product was measured at 405 nm. The conditioned medium from the cells treated with medium only was used as a negative control.

### Ca^2+^ Measurement

The B16F10 tumor cells were seeded in 96-well plates. At approximately 80% confluence, the cells were treated with hGAG (0–1.0 µM) for 24 h/37°C, the medium was then removed and 100 µl of DMEM/10% FBS containing 3.6 µM fluo-4AM was added. Cells were further incubated for 30 minutes at 37°C/5% CO_2_, followed by adding 200 µl/well of Hanks balanced salt solution without phenol. Fluorescence was measured after excitation at 485 nm and emission at 520 nm using a microplate fluorometer (Sunrise, Switzerland). Results were expressed as relative changes in fluorescence after the addition of hGAG *vs* control [33]. Cells treated with medium only were used as a negative control.

### Phosphatidylserine (PS) Exposure Assay

The extent of PS exposure in the hGAG treated B16F10 cells were assessed using the cell surface labeled Annexin V-FITC method as previously described [34], [35]. In brief, B16F10 cells were seeded into the 6-well plates and incubated overnight, followed by treatment with hGAG (0–1.0 µM) for 24 h/37°C. After washing in ice-cold PBS and trypsinization, cells were resuspended in binding buffer (2%FBS/PBS, 0.1%NaN_3_) at a concentration of 10^6^ cells/ml. An aliquot of 0.5 ml was transferred to a culture tube and incubated with 5 µl FITC-conjugated Annexin V (BD Biosciences, San Diego, CA) at room temperature for 10 min in dark. After removal of supernatant by centrifugation, B16F10 cells were resuspended in 500 µl cold binding buffer, followed by the addition of 10 µl PI and incubated on ice for 15 min in dark. Cells were immediately processed for fluorescence-activated cell sorting (FACS) using BD FACSCalibur flow cytometer (BD Biosciences, San Jose, CA). Flow cytometry data were analyzed using CellQuest software (BD Biosciences) and interpreted as a mean number of events in each quadrant.

### Western Blot

Total protein extraction, separation and transfer were performed according to the established protocol [36]. The membranes were blocked in 5% milk and incubated overnight at 4°C with the primary antibody (*e.g*., TF, p38, p-p38, ERK, p-ERK, JNK, and p-JNK *etc*.) as indicated in the text. After incubation with a HRP-conjugated secondary antibody for 1 hour at room temperature, and chemiluminescent signals were visualized using the enhanced chemiluminescence (ECL) reagent (Millipore Corpaoration, Billerica, MA). GAPDH was used as a loading control.

### Quantitative Real-time PCR (qPCR)

Total mRNA was extracted using the Trizol reagent according to the manufacturer’s instructions, and 1 µg of total RNA was used for the synthesis of the first strand cDNA [36]. The expression of TF was analyzed by qPCR with SYBR Green I Master using a LightCycler® 480 Real-time PCR system (Roche Applied Science, Penzberg, Germany). TF was amplified using its specific primers (sense: 5′-CATGG AGACG GAGAC CAACT-3′; antisense: 5′-CCATC TT GTT CAAAC TGCTG A-3′) as described [37]. The level of β-actin (sense: 5′-GAGAA GATCT GGCAC CACAC C-3′; antisense: 5′-GCATA CAGGG AC AGC ACAGC-3′) was used as an internal control.

### Promoter Deletion and Luciferase Assay

TF promoter truncation was performed by PCR from mouse genome to delete single transcriptional factor binding site for each construct [38]. In total, 5 different fragments were amplified: namely, (*i*) full length of TF promoter; (*ii*) a fragment (−278–+121) including the binding sites of four transcriptional factors (AP-1, NF-κB, Sp1, and Egr1); (*iii*) a fragment (−197–+121) with AP1 deletion); (*iv*) a fragment (−175–+121) with AP-1 and NF-κB deletions; and (*v*) a fragment (−154–+121) with AP-1, NF-κB and Sp1 deletions. The PCR amplified products were cloned into PMD-18T, and then subcloned into the pGL3 enhancer plasmid to respectively generate expression vectors: (*i*) pTF-Luc, (*ii*) pTF/5′-d-Luc, (*iii*) pTF/AP-d-Luc, (*iv*) pTF/AN-d-Luc, and (*v*) pTF/ANS-d-Luc, as depicted in the text. All the recombinant plasmids were confirmed by sequencing to be identical to those in Genbank (GI: 201924 for murine TF). The constructed vectors were used to transfect B16F10 cells for 48 h using Lipofectamine™ 2000 (Invitrogen) according to the manufacturer’s protocols. The transfected B16F10 cells were then treated with different concentrations of hGAG for 24 h/37°C and the cells were lysed with passive lysis buffer (Promega). Luciferase activity was measured using Luciferase assay kit (Promega, Madison, WI). B16F10 cells treated with medium only were used as control.

Mutagenesis of NF-κB binding site (from 5′-CGGAG TT**TCC** TAC-3′ to 5′-CGGAG TT**AAA** TAC) in the promoter fragment (−278–+121) in pTF/5′-d was done using the MutanBEST Kit (Takara Sake, Berkeley, CA) and was confirmed by sequencing. All the plasmid construction, cell transfection, hGAG treatment, and luciferase assay were conducted using the similar methods as described above.

### Cell Migration

Cell migration was assessed using wound healing and Transwell assays as previously described [36]. Briefly, for wound healing assay, monolayer cells at 90–95% confluence were serum starved for 24 h and then carefully wounded using sterilized pipette tips (t = 0 h). After removing detached cells, the cells were incubated with medium, tumor necrosis factor (TNFα, final conc. 50 nM), or TNFα in combination with hGAG at the indicated concentration for 24 h at 37°C and photographed immediately (t = 24 h).

### Data Analysis

All the experiments were performed in triplicates and the quantitative data are expressed as mean ± SD. Comparisons were analyzed by Student’s *t*-test and ANOVA with GraphPad Prism 5 (GraphPad Software Inc., San Diego, CA). *p*<0.05 was considered significant difference.

## Results

### hGAG Treatment Decreased Metastasis of B16F10 Tumor Cells and Coagulation Capacity of Plasma *in vivo*

To investigate whether hGAG could play a critical role in preventing metastasis and thrombosis *in vivo*, the metastatic B16F10 tumor cells were treated with hGAG at different concentrations (0–1.0 µM) for 24 h/37°C. The hGAG-treated B16F10 tumor cells and the cells treated with medium only (as control) were injected into the mice through tail veins and the lung tissues were collected on day 23 for metastatic evaluation. As indicated in Figure 1B, the melanin nodules that were observed on the surface of the lung tissues were significantly reduced with the increase of hGAG treatment. Notably, the B16F10 tumor cells treated with 1 µM of hGAG generated much less melanoma nodules compared to the cells treated with medium only (*p*<0.01, Fig. 1B, lower panel). Furthermore, in the control group, HE staining showed high amount of metastatic tumor cells in the lung tissue section (Fig. 1C, a). In contrast, the metastatic capacity was significantly reduced in lung tissues from the hGAG-treated groups (Fig. 1C, b–d). Clearly, the *in vivo* data indicate that hGAG shows a remarkable inhibitory effect on metastasis. Because cell migration plays a crucial role in determining metastatic capacity of cancer cells [39], we then examined the effect of hGAG on cell migration using both the Wound Healing (Fig. 1D) and Transwell (Fig. S1) assays. As indicated in Figure 1D, hGAG did not inhibit TNF-induced migration of the B16F10 tumor cells. This was substantiated by studies using the Transwell assay (Fig. S1). Thus, the hGAG-inhibited metastasis was not due to its inhibitory effect on cell migration.

hGAG demonstrated significant antithrombotic efficacy in mouse [23] and rat models [24], we next examined whether hGAG could affect the coagulation capacity of the blood in the presence of hGAG-treated tumor cells *in vivo*. Given that TF physiologically initiates blood coagulation and correlates with metastatic potential [4], [5], the TF level was assessed by ELISA from the blood samples collected on day 7, day 14 and day 23, respectively. As indicated in Figure 1E, the level of TF in the plasma was significantly decreased in the hGAG-treated groups in a dose dependent manner, relative to the control group (*p*<0.05–0.01). In addition, the levels of activated partial thromboplastin time (APTT), prothrombin time (PT) and thrombin time (TT) were also measured to evaluate the coagulation capacity. We showed that the three types of coagulation times were generally extended in the hGAG-treated groups compared to the control group (*p*<0.05) (Fig. S2). Hence, the *in vivo* studies reinforced the antithrombotic function of hGAG.

### B16F10 Cells Treated with hGAG Reduced the Formation of Fibrin Rather than Increased Fibrinolysis

**Figure 2:**
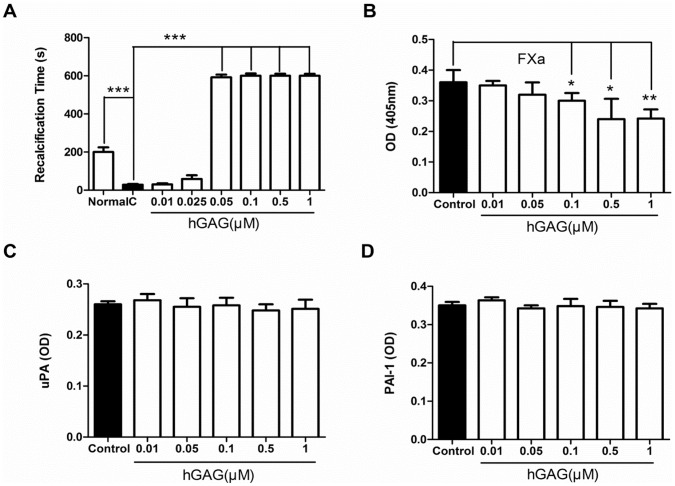
Effects of hGAG on B16F10 tumor cells-initiated fibrin formation and fibrinolysis. (**A**) Coagulation time. Addition of B16F10 tumor cells to the normal plasma of mice significantly decreased the coagulation time, compared to the normal plasma only, indicating an efficient coagulation capacity of tumor cells. Treatment of cells with hGAG remarkably increases the coagulation time, implicating a reduced coagulation capacity of hGAG-treated cells. (B) hGAG treatment inhibits generation of activated FXa. B16F10 tumor cells were treated with hGAG (0–1.0 µM) for 24 h/37°C and the conditioned medium was used for assessing FXa activity. Conversion of FXa substrate S2222 to a chromogenic product was measured at 405 nm. (C) uPA and (D) PAI-1 levels produced by B16F10 tumor cells in presence or absence of hGAG. The uPA and PAI-1 levels were assayed by ELISA. Data represents as mean ± S.E. (3–5 independent experiments). *p<0.05, **p<0.01, ***p<0.001.

The *in vivo* studies established the inhibitory effect of hGAG on metastasis of B16F10 tumor cells and plasma coagulation. To substantiate this function *in vitro*, the B16F10 tumor cells were treated with hGAG (0–1.0 µM) for 24 h/37°C and then the normal mice plasma were added. We showed that addition of B16F10 tumor cells to the plasma significantly reduced the coagulation time (*p*<0.001) compared to the normal plasma alone (Fig. 2A), indicating a significant coagulation capacity of these cells. Nevertheless, relative to the tumor cells treated with medium only, the coagulation times were remarkably increased when the cells were treated with >0.05 µM of hGAG (*p*<0.001) (Fig. 2A). Therefore, hGAG treatment *in vitro* resulted in a significant decrease of the tumor cells-induced coagulation.

Since the coagulation is determined by two processes: (*i*) decreasing the fibrin generation and, (*ii*) increasing the fibrinolysis [40], [41], we then examined which step is predominantly affected by hGAG treatment. Fibrin formation involves two routine coagulation cascades: intrinsic coagulation cascade and extrinsic coagulation cascade [42] and the FXa is a critical protein regulating fibrin formation [40]. Considering this, we analyzed the level of FXa in the conditioned medium produced from both the control and hGAG-treated cells. As shown in Figure 2B, hGAG treatment decreased the generation of FXa in a dose dependent manner, with a significant reduction (*p*<0.05–0.01) when the cells were treated with >0.1 µM hGAG, compared to the control cells treated with medium only. On the other hand, assessing the levels of uPA and PAI-1 which are two main factors for fibrinolysis [40], we found both the hGAG-treated cells and control cells expressed similar levels of uPA (Fig. 2C) and PAI-1 (Fig. 2D) by ELISA. These data indicate that the hGAG-induced increase of coagulation time was not a consequence of fibrinolysis. Instead, hGAG treatment reduced the fibrin generation *via* inhibiting FXa.

### hGAG Inhibits FXa Generation through Suppressing TF and Decreasing PS Exposure

**Figure 3:**
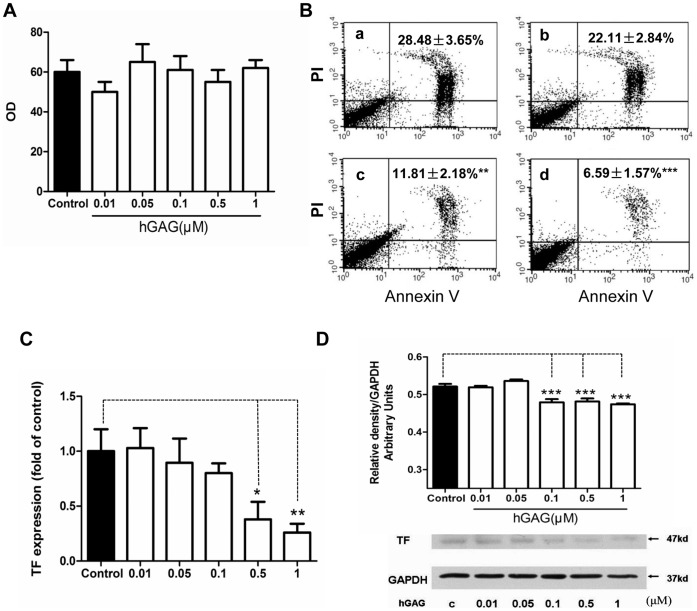
Effects of hGAG on Ca^2+^ efflux, phosphatidylserine (PS) exposure and TF expression. (A) hGAG treatment shows no effect on Ca^2+^ efflux. B16F10 tumor cells were treated with hGAG (0–1 µM) for 24 h/37°C and fluo-4AM was used to measure the level of Ca^2+^. (B) hGAG treatment decreases the extent of phosphatidylserine exposure in the B16F10 tumor cells. Cells were treated with hGAG at the indicated concentration (**a**: medium only; **b**: hGAG 0.01 µM; **c**: hGAG 0.1 µM; **d**: hGAG 1.0 µM) and stained with Annexin V-FITC and PI (nucleus). Note that with the increase of hGAG concentration, the percentage of cells displaying Annexin-FITC signals was significantly reduced compared to the cells treated with medium only (a). (C) hGAG treatment inhibits TF transcription in the B16F10 tumor cells. Relative mRNA levels were assayed by quantitative real-time PCR and expressed after normalized to the control samples. (D) hGAG treatment reduces TF protein expression. B16F10 tumor cells were treated with hGAG at the indicated concentration for 24 h and the level of TF was analyzed by western blotting. GAPDH was used as a loading control. Data are represented as mean ± S.E. *p<0.05, **p<0.01, ***p<0.001.

FXa generation requires three basic elements, namely Ca^2+^, PS exposure and TF [40]. We showed that Ca^2+^ efflux, an indispensable element during the coagulation process, appeared to have no significant differences between the hGAG-treated groups and control group (Fig. 3A). Given that PS exposure can provide a phospholipids surface for the assembly of enzyme complex, *e.g*., TF/FVIIa, on the cell surface [43], the extent of PS exposure can be assessed by evaluating the signals stained with Annexin V-FITC [34]. In the cells treated with medium only (Fig. 3B, a), approximately 28.48±3.65% of B16F10 cells displayed Annexin V-FITC positive signals. In contrast, with the increase of hGAG treatment (0.01, 0.1 and 1.0 µM), the percentage of cells that showed Annexin V-FITC signals was significantly reduced (*e.g*., 28.48±3.65% *vs* 6.95±1.57%, *p*<0.001) (Fig. 3B, b–d). These data suggest that hGAG treatment could facilitate an inhibition of PS exposure and this inhibition occurs in a dose dependent manner (Fig. 3B).

We next examined whether hGAG can regulate TF expression. To this end, B16F10 cells were treated with different concentrations of hGAG (0–1.0 µM) for 24 h/37°C and TF transcription levels were assessed by qPCR. As demonstrated in Figure 3C, hGAG treatment remarkably decreased the mRNA expression of TF when the cells were treated with >0.5 µM of hGAG (*p*<0.05–0.01) *versus* cells treated with medium only. This was further substantiated by immunoblot analysis showing that, compared to the control, hGAG significantly reduced TF protein expression (Fig. 3D). Collectively, hGAG treatment markedly reduces both the mRNA and protein expressions of TF.

### Nuclear Transcriptional Factor NF-κB is Responsible for hGAG-induced Suppression of TF Transcription

**Figure 4:**
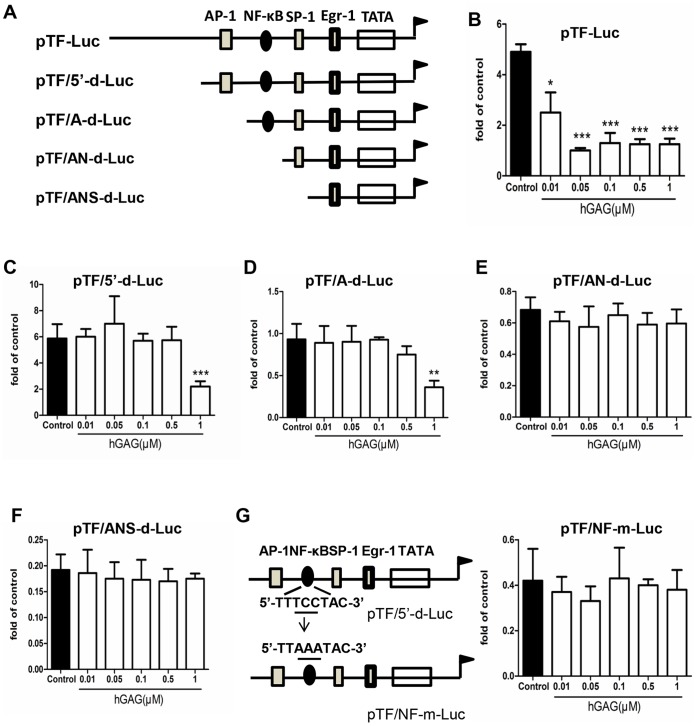
TF promoter deletion analysis. (A) Schematic structure of TF promoter fused with Luciferase expression gene. hGAG effect on luciferase activity of B16F10 cells transfected with pTF-Luc (B), pTF/5′-d-Luc (C), pTF/A-d-Luc (D), pTF/AN-d-Luc (E) pTF/ANS-d-Luc (F). B16F10 cells were transfected with the constructed luciferase expression vector and treated with hGAG at the indicated concentration for 24 h. Luciferase activity was assayed. (G) Mutation of NF-κB binding site. Mutation of NF-κB binding site was performed using MutanBEST Kit (Takara) and the mutated pTF/NF-m-Luc was transfected into B16F10 cells. hGAG treatment and luciferase assay were carried out following the same procedure as above. Data are represented as mean ± S.E. from 3–5 independent experiments. *p<0.05, **p<0.01, ***p<0.001.

To understand the molecular mechanisms underlying hGAG-mediated inhibition of TF, the full length or truncated fragments of TF promoter were generated and fused into the luciferase reporter vector (Fig. 4A). To identify the candidate transcription factors that could respond to hGAG, these generated plasmids (Fig. 4A) were transiently transfected into B16F10 cells, followed by treatment with hGAG (0–1.0 µM) for 24 h/37°C. We found that in the B16F10 cells transfected with pTF containing full length TF promoter, the luciferase activity was markedly decreased with the increase of hGAG (Fig. 4B). Interestingly, the cells transfected with pTF/5′-d resulted in a loss of sensitivity to hGAG treatment at low concentrations, and a significant reduction of luciferease activity was only occurred when the cells were treated with hGAG at 1 µM (Fig. 4C). This may indicate a deletion of potential enhancer elements at the 5′-termini. While deletion of AP-1 binding site in the plasmid (pTF/A-d) led to a similar response of luciferase activity to hGAG treatment (Fig. 4D) as observed in the cells transfected with pTF/5-d (Fig. 4C), further deletion of NF-κB site (pTF/AN-d) resulted in a completely loss of luciferase activity in B16F10 cells treated with hGAG at 1 µM (Fig. 4E), and SP-1 deletion (pTF/ANS-d) showed a similar result (Fig. 4F). These data indicate that NF-κB binding site is critical for the hGAG-inhibited TF transcription.

To further verify the significance of NF-κB binding site, the conserved sequence of NF-κB binding element, 5′- TT**TCC** TAC-3′, was mutated to 5′-TT**AAA** TAC-3′ in pTF/5′-d and the generated vector pTF/NF-m (Fig. 4G) was transiently transfected to B16F10 cells. As indicated in Figure 4G and compared to Figure 4C, mutation of this conservative region led to a completely loss of response to the hGAG treatment based on the luciferase activity. This further verified the significance of NF-κB binding site that responds to hGAG treatment.

### hGAG Regulates NF-κB Transclocation, but not Expression, in B16F10 Cells

**Figure 5:**
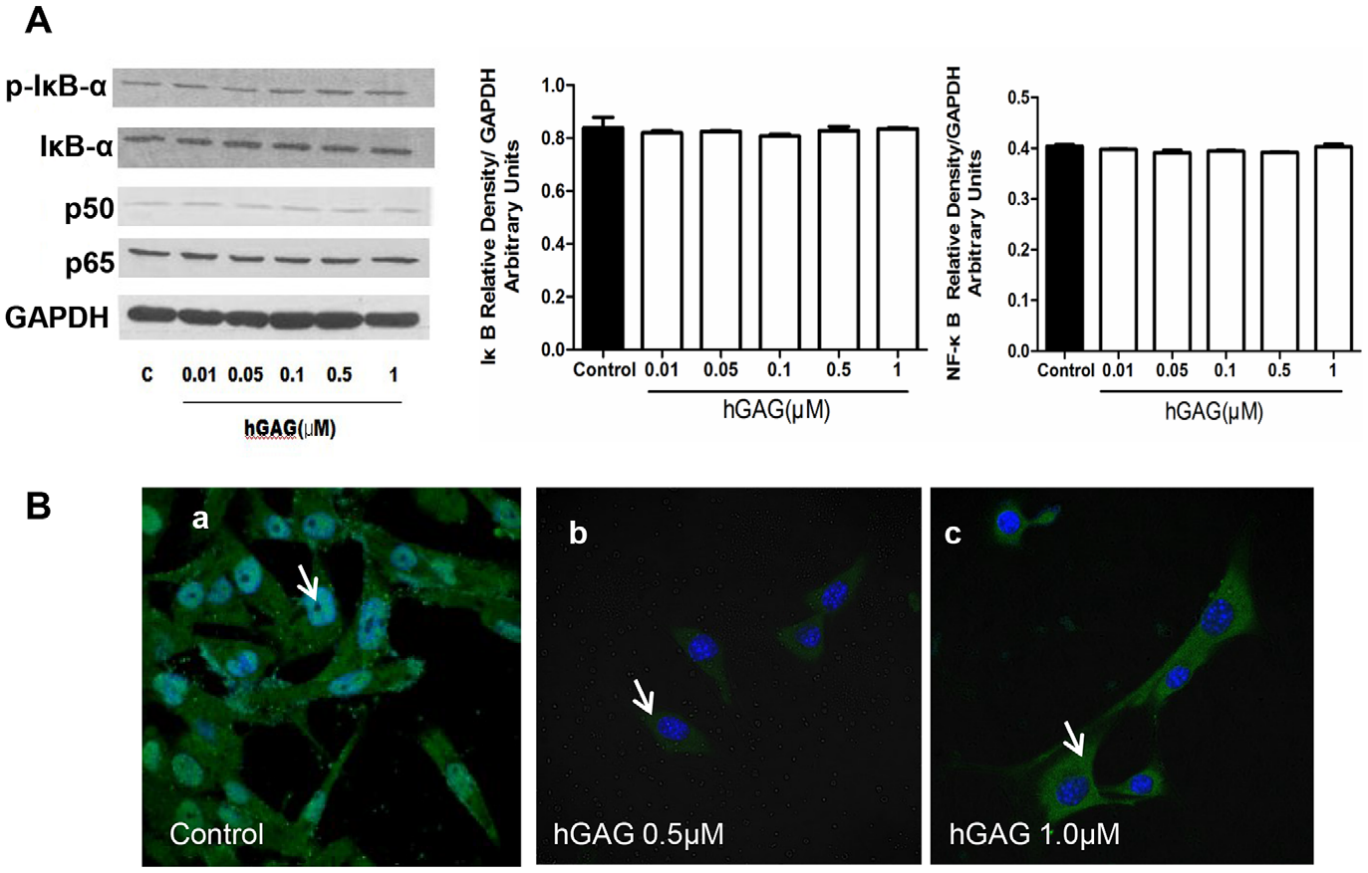
Effects of hGAG on NF-κB signaling. (A) hGAG treatment shows no effect on the expression of NF-κB(p65), NF-κB(p50) and its inhibitor IκBα in B16F10 tumor cells. Data are represented as mean ± S.E. from 3–5 independent experiments. (B) hGAG treatment blocks nuclear translocation of NF-κB(p65). B16F10 cells were seeded on coverslip and treated with hGAG at 0, 0.5 and 1.0 µM for 24 h/37°C. Immunofluorescence (IF) staining of NF-κB(p65) was performed. Note the nuclear staining of NF-κB(p65) in the control (**a**) and predominantly cytoplasmic staining in the hGAG-treated cells (**b** and **c**). Representative IF is shown from 3–5 experiments.

The above studies showed that hGAG treatment downregulated the TF/FXa pathway (Fig. 2B and 3) and this was mediated *via* affecting TF transcription factor NF-κB (Fig. 4). However, it is uncertain whether hGAG exerts its function by reducing NF-κB expression or by affecting its nuclear localization. To examine this, the expressions of NF-κB(p65) and NF-κB(p50), and NF-κB inhibitor Iκβα and p-Iκβα were analyzed. As shown in Figure 5A, hGAG treatment did not significantly affect expression of these molecules compared to the control cells treated with medium only. Instead, when the B16F10 cells were treated with hGAG at 1 µM, the immunofluorescence analysis revealed that NF-κB(p65) was predominantly observed in the cytosol compared to the control cells in which NF-κB(p65) was relative evenly localized in both nucleus and cytosol (Fig. 5B). Hence, hGAG down-regulates TF expression *via* blocking nuclear translocation of NF-κB.

### hGAG Suppresses MAPK Signaling in B16F10 Cells

It has been demonstrated that the PI3K/p-Akt [9], [10] and MAPK pathways [11], [12] regulated metastasis that was mediated through the nuclear NF-κB-regulated MMP expression and secretion [13]–[16]. To elucidate the regulatory effect of hGAG on these known signaling pathways in B16F10 tumor cells, the activation of AKT, Jak2, Stat3, FAK, Smad2 and MAPK proteins (p38, p-p38, ERK, p-ERK, JNK and p-JNK) were assessed in the cells treated with hGAG for 24 h/37°C at the indicated concentrations. We demonstrated that hGAG treatment did not show any significant effects on the activation/phosphorylation of Jak/Stat3 (Fig. S3A), a central cascades mediating signal transduction from cytokine receptors [44], [45], and of FAK and Smad2 that regulate cell migration and metastasis [46], [47] (Fig. S3B). Similarly, hGAG treatment was unable to affect p-AKT expression, indicating that PI3K/AKT pathway was not regulated by hGAG (Fig. S3C). Again, the expression and phosphorylation of both of the upstream regulator, p70S6K1, and the downstream effector, GSK, of AKT signaling [48] were not affected by hGAG (Fig. S3D).

**Figure 6:**
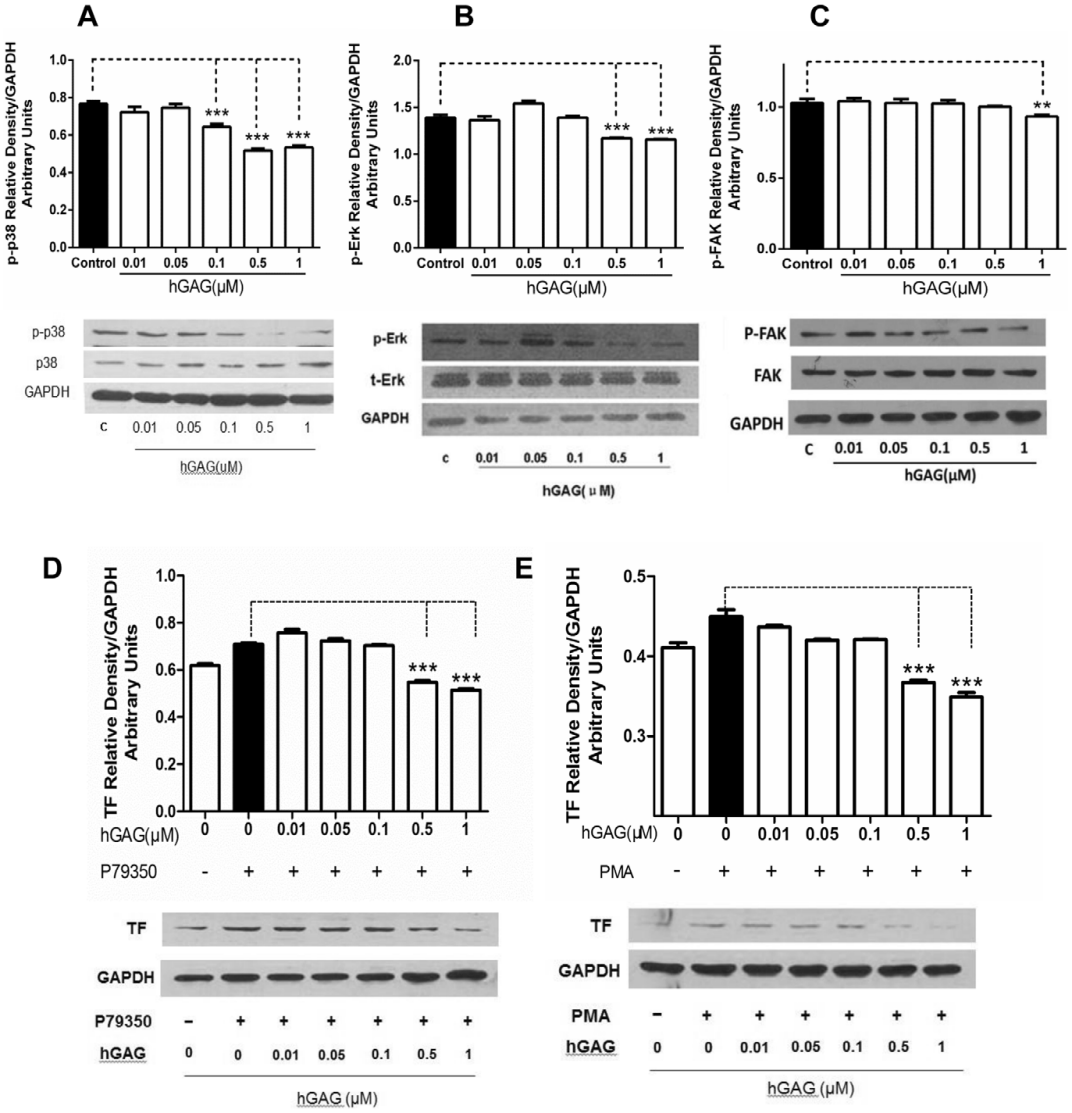
hGAG suppresses p38 and ERK signaling to inhibit TF expression in B16F10 tumor cells. hGAG significantly inhibits p38 phosphorylation at >0.1 µM (A), ERK phosphorylation at >0.5 µM (B) and FAK phosphorylation at 1.0 µM (C). (D) hGAG treatment reverses p38 agonist P79350-induced TF expression. B16F10 tumor cells were treated with P79350 (50 µM) alone or in combination with hGAG at the indicated concentration for 24 h/37°C. TF expression was assessed by western blot. Note the P79350-induced TF expression was remarkably decreased by hGAG at >0.5 µM. (E) hGAG treatment represses ERK agonist PMA-induced TF expression. B16F10 tumor cells were treated with PMA (10 µM) alone or in combination with hGAG at the indicated concentration for 24 h/37°C. PMA-induced expression of TF in B16F10 tumor cells was significantly decreased by hGAG at >0.5 µM. All data are represented as mean ± S.E. from 3–5 independent experiments. **p<0.01 ***p<0.001.

Nevertheless, while the basal levels of ERK and JNK were not markedly affected by hGAG treatment, p38 expression was gradually enhanced with the increase of hGAG (Fig. 6A). Notably, phosphorylations of ERK and p38 were decreased in a dose-dependent manner, with a significant (*p*<0.05) reduction of p-p38 and p-ERK1/2 at >0.5 µM and p-FAK at 1.0 µM of hGAG (Fig. 6A–C). These data indicate that hGAG exerts its inhibitory function to NF-κB/TF/FXa *via* selectively suppressing the activation of MAPK pathway. To further substantiate the involvement of p-p38 and p-ERK in this process, the B16F10 tumor cells were treated with p-p38 agonist P79350 at 50 µM alone or in combination with hGAG at the indicated concentration for 24 h/37°C. P79350 has been used to specifically activate p38 signaling in rat spinal ligament cells [49]. As demonstrated in Figure 6D, cells treated with P79350 showed a significant increase of TF expression compared to the vehicle treated (0.1% DMSO) control cells (*p*<0.05). Addition of hGAG at >0.5 µM reverses the activated p-p38 induced TF expression (*p*<0.001). Similarly, considering that PMA could induce activation of protein kinase C (PKC)/mitogen-activated protein kinase kinase (MEK)/ERK pathway [50], [51], the B16F10 tumor cells were treated with PMA at 10 µM alone or in combination with hGAG at the indicated concentration for 24 h/37°C and the TF expression was analyzed. Likewise, cells treated with PMA alone displayed an enhanced expression of TF, and hGAG treatment at >0.5 µM significantly attenuated TF expression facilitated by ERK activation. Taken together, our data demonstrate that hGAG suppresses NF-κB/TF/FXa through repressing p-p38 and p-ERK pathways.

## Discussion

hGAG has been demonstrated as a novel and safe therapeutic agent for metastatic inhibition and antithrombosis [23], [25]. However, the relevant mechanism underlying these functions was not previously defined. In this study, we reported that hGAG can suppress cancer cell metastasis and TF/FXa pathway by repressing activation of MAPKs (p38MAPK and ERK1/2)/NF-κB pathway.

Cancer is a disease that associates with venous thrombosis. Cancer patients with thrombosis have a worse prognosis due to the fact that cancer cells directly contribute to the hypercoagulable state [1], [2]. Recently, considerable effort has been made to identify novel therapeutic agents with both anti-cancer and antothrombosis activity. The hGAG, a bioactive natural product isolated from the sea cucumber [22], has been demonstrated to be a promising drug that showed a significant anti-metastatic and anti-thrombotic function [23]–[25]. In support of this, we also demonstrated that the metastatic efficiency of B16F10 melanoma cancer cells to the lung tissues was remarkably suppressed by hGAG. Meanwhile, the blood samples from the mice injected with hGAG-treated cells displayed a reduced coagulation activity, as assessed by measuring APTT, PT and TT. Intriguingly, hGAG significantly repressed the expression of TF in the B10F16 cells circulated in blood. Because TF expression in cancer cells has been shown to correlate with metastatic potential [52], [53] and the formation of pathological clots or thrombi in the veins of cancer patients [54], the reduction of TF expression might be a mechanism by which hGAG suppresses both metastasis and thrombosis in B16F10 cells.

The interactions between metastatic cancer cells and blood coagulation system are a complicated biochemical process that involves the expression of cell surface proteins and assembly with coagulation factors from blood. In general, the coagulation cascade includes two distinct pathways: the intrinsic pathway and extrinsic pathway [42], and the presence of tumor cells, inflammation and injury can initiate the extrinsic coagulation cascade [55]. Notably, TF, as a cell surface glycoprotein, is responsible for initiating the extrinsic pathway of coagulation [56] by binding with the plasma cofactor Factor VII (FVII) and activating FX in the presence of Ca^2+^ and exposure of PS. The activated FXa then initiates the clotting cascade, leading to the generation of thrombin from prothrombin and ultimately fibrin clot [33]. In our studies, we demonstrated that while the hGAG treatment did not affect Ca^2+^ status, the extent of PS exposure was decreased in the cells treated with hGAG. This may indicate a possible reason for the hGAG-mediated inhibition of FX activation. Considering that hGAG treatment reduced the formation of fibrin, a factor that protects cancer cells from the natural killer cell-mediated elimination in blood [57], inhibition of TF/FXa pathway by hGAG could play a key role in this process in our cell model. Consequently, the inhibition of procoagulant capacity of B16F10 cancer cell by hGAG is mediated by the loss of TF expression and PS exposure.

It has been demonstrated that the transcription factors AP-1, NF-κB, Sp1 and Egr-1 can directly bind to TF promoter to regulate its constitutive expression [38]. Here we found that NF-κB is the key player in regulating hGAG-inhibited TF transcription *via* a mechanism involved in decreased nuclear translocation of NF-κB. It has been demonstrated that NF-κB plays a key role in regulating TF expression [38]. In addition, the specific inflammatory mediators including TNFα, interleukin-1 (IL-1) and lipopolysaccharide (LPS) increased TF transcription through activating the transcription factor NF-κB and/or AP-1 [58], [59]. Because NF-κB activation has been involved in many types of cancer metastasis that are likely induced by its downstream pro-inflammatory cytokines such as IL6 and GM-CSF [60]–[62], targeting NF-κB and its associated pathways may open a possibility for therapeutic intervention of metastatic cancer.

Previous studies supported a significant link of the PI3K/Akt, Jak/Stat, FAK, TGFβ/Smad and MAPKs (*e.g*., JNK, ERK1/2 and p38) with cancer cells proliferation, invasion, and metastasis for a wide range of tumors [11], [63], [64]. However, in our studies, hGAG treatment did not affect activation of Akt, Jak/Stat, JNK and Smad pathways in our cell model. Instead, the phosphorylations of ERK1/2 and p38 were significantly inhibited by hGAG in a dose dependent manner. Our data suggest that hGAG can specifically target MAPKs to suppress their activation. It was reported that MAPK pathways play a critical role in regulating the expression of MMPs by activating NF-κB [65], [66]. This may provide a molecular basis underlying the hGAG-mediated inactivation of NF-κB/TF and repression of *in vivo* metastasis of B16F10 cells. Our findings proved that hGAG-mediated inhibition of metastasis and procoagulant activity of B16F10 cancer cells involve MAPKs (p38 MAPK, ERK1/2)/NF-κB/TF signaling pathways.

In summary, cancer metastasis is facilitated by the hematogenous spread of CTCs that have played significant roles in the association of cancer cells and thrombosis. While most of the CTCs are killed by the natural killer cells during circulation in blood vessels, the CTCs-associated activation of coagulation cascades enable them to escape from the surveillance of immune system, and subsequently metastasize to distant sites [57], [67]. The efficiency of hGAG in suppressing *in vivo* metastasis of B16F10 cells through the disruption of cell-blood interactive system may provide new insight into therapeutic interventions for metastatic disease.

## Supporting Information

Figure S1 Effect of hGAG treatment of B16F10 tumor cells on TNFα-induced cell migration. B16F10 tumor cells (7.5×10^5^) were treated with vehicle control (A), TNFα alone (B) or in combination with hGAG (C–E) for 24 h and cell migration was investigated using transwell system. Migrated cells were stained and photographed. Arrows indicate the migrated cells attached onto membrane. Note that hGAG shows no significant inhibitory effect on the TNF α -induced migration.
(TIF)

Figure S2 Assessment of HGAG treatment on the B16F10 tumor cells-mediated activated partial thromboplastin time (APTT), prothrombin time (PP) and thrombin time (TT) *in vivo*. B16F10 tumor cells treated with medium alone or hGAGA at the indicated concentrations were injected into mice through tail vein, Blood samples were taken on day 7, 16 and 23 for assessing the activated partial thromboplastin time (APTT), prothrombin time (PP) and thrombin time (TT). Compared to the normal blood from the mice without injecting tumor cells, the blood sample from the mice injected with tumor cells showed a significant decrease of PT, APTT and TT. However, the blood samples taken from the mice injected with hGAG-treated tumor cells showed an increase of PT, APTT and TT, compared to those from the control. *p<0.05, **p<0.01, ***p<0.001.
(TIF)

Figure S3 Effect of hGAG on the activation of Jak/Stat, JNK, Smad2, Akt and GSK3β. B16F10 tumor cells were treated with medium or hGAG at the indicated concentrations for 24 h/37°C and the expression of proteins were analyzed by western blot. Note that hGAG has no effect on activation of pathways relevant to these molecules.
(TIF)
