# Supplementary figures and images for: Holothurian Glycosaminoglycan Inhibits Metastasis and Thrombosis via Targeting of Nuclear Factor-κB/Tissue Factor/Factor Xa Pathway in Melanoma B16F10 Cells
Source: PLoS One. 2013 Feb 21;8(2):e56557. doi: 10.1371/journal.pone.0056557 (PMC3578936; doi:10.1371/journal.pone.0056557)

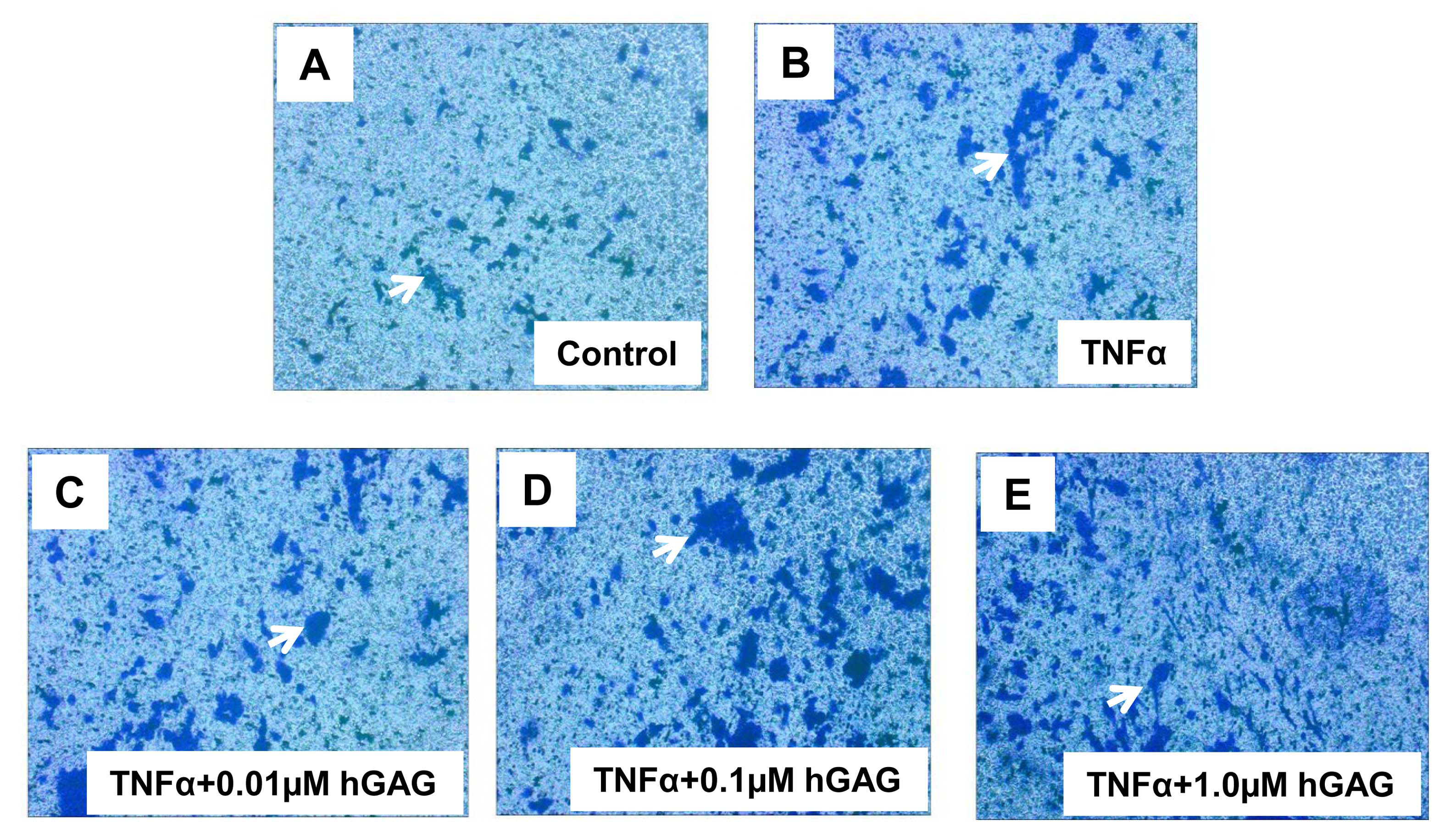

Supplement: Figure S1 — Effect of hGAG treatment of B16F10 tumor cells on TNFα-induced cell migration. B16F10 tumor cells (7.5×10^5^) were treated with vehicle control (A), TNFα alone (B) or in combination with hGAG (C–E) for 24 h and cell migration was investigated using transwell system. Migrated cells were stained and photographed. Arrows indicate the migrated cells attached onto membrane. Note that hGAG shows no significant inhibitory effect on the TNF α -induced migration. (TIF) [file pone.0056557.s001.tif]

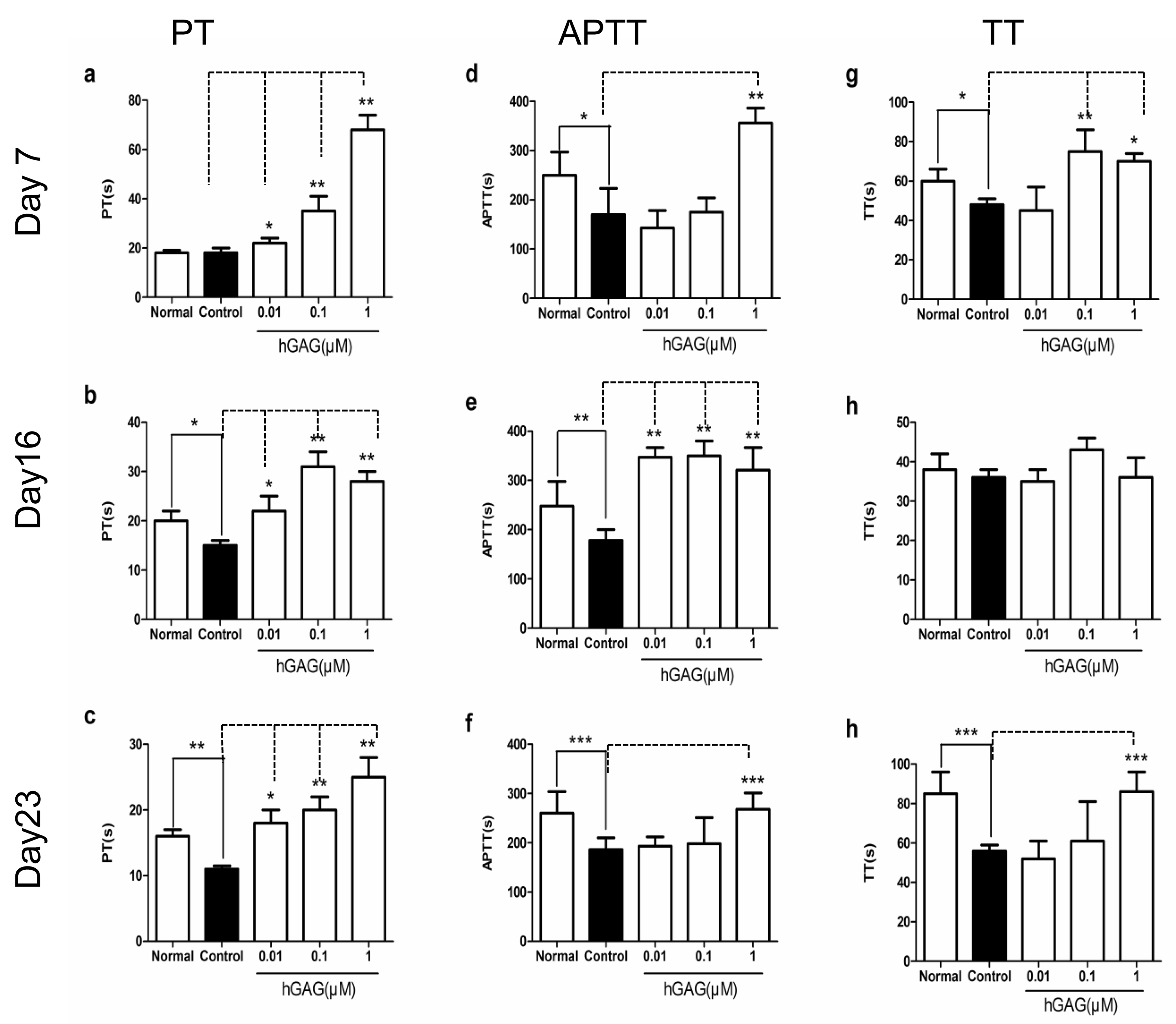

Supplement: Figure S2 — Assessment of HGAG treatment on the B16F10 tumor cells-mediated activated partial thromboplastin time (APTT), prothrombin time (PP) and thrombin time (TT) in vivo. B16F10 tumor cells treated with medium alone or hGAGA at the indicated concentrations were injected into mice through tail vein, Blood samples were taken on day 7, 16 and 23 for assessing the activated partial thromboplastin time (APTT), prothrombin time (PP) and thrombin time (TT). Compared to the normal blood from the mice without injecting tumor cells, the blood sample from the mice injected with tumor cells showed a significant decrease of PT, APTT and TT. However, the blood samples taken from the mice injected with hGAG-treated tumor cells showed an increase of PT, APTT and TT, compared to those from the control. *p<0.05, **p<0.01, ***p<0.001. (TIF) [file pone.0056557.s002.tif]

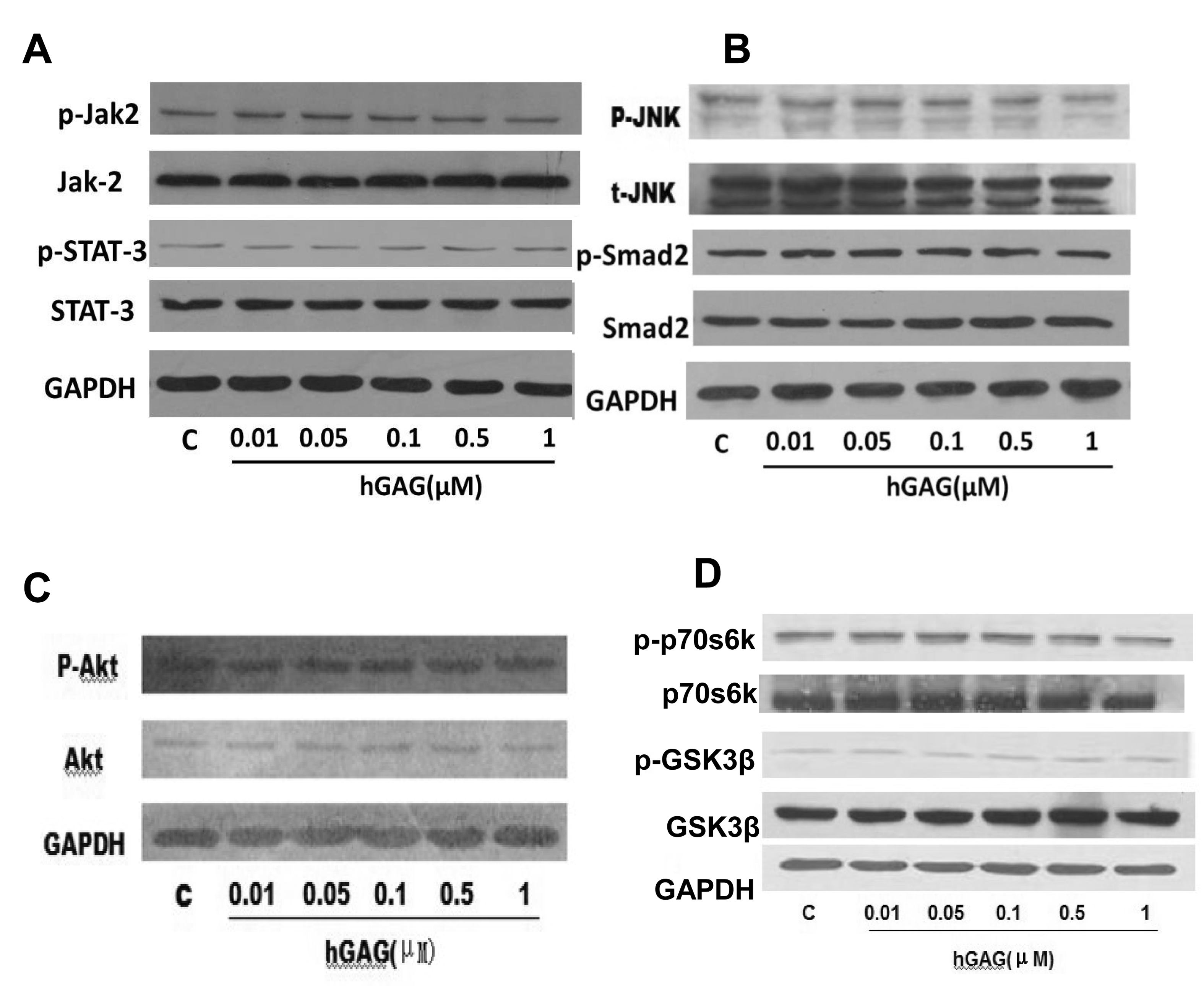

Supplement: Figure S3 — Effect of hGAG on the activation of Jak/Stat, JNK, Smad2, Akt and GSK3β. B16F10 tumor cells were treated with medium or hGAG at the indicated concentrations for 24 h/37°C and the expression of proteins were analyzed by western blot. Note that hGAG has no effect on activation of pathways relevant to these molecules. (TIF) [file pone.0056557.s003.tif]
